# Evaluating HER2 Scoring Criteria in Endometrial Carcinoma: Gynecologic Versus Gastric Guidelines for Trastuzumab and Trastuzumab-Deruxtecan Selection

**DOI:** 10.3390/cancers18122009

**Published:** 2026-06-22

**Authors:** Sharon Nofech-Mozes, Ekaterina Olkhov-Mitsel, Fang-I Lu, Weei-Yuarn Huang, Anna Plotkin

**Affiliations:** 1 Precision Diagnostics and Therapeutics Program, Division of Anatomic Pathology, Department of Laboratory Medicine and Molecular Diagnostics, Sunnybrook Health Sciences Centre, Toronto, ON M4N 3M5, Canadaweeiyuarn.huang@sunnybrook.ca (W.-Y.H.); 2 Department of Laboratory Medicine and Pathobiology, Temerty Faculty of Medicine, University of Toronto, Toronto, ON M5S 3G9, Canada

**Keywords:** endometrial carcinoma, *ERBB2* amplification, HER2 immunohistochemistry, biomarker testing, targeted therapy

## Abstract

Background/Objectives: HER2 overexpression and/or amplification defines a molecularly distinct subset of endometrial carcinomas (ECs) that may benefit from HER2-targeted therapies. However, HER2 testing algorithms remain non-standardized and vary across institutions. This study is a large single-institution audit of EC HER2 testing practices, using both gynecologic (ISGyP) and gastric cancer-specific scoring algorithms at a major academic center with a reference gynecologic oncology service and biomarker laboratory. Methods: HER2 immunohistochemistry (IHC) and whole-slide fluorescence in situ hybridization (FISH) were interpreted by subspecialty breast and gynecologic pathologists, with HER2 IHC performed on 494 tumor samples (2021–2025) and reflex FISH for equivocal cases. Results: Using ISGyP criteria, 15.0% (74/494) of tumors were HER2 IHC 3+, 44.5% (220/494) equivocal (2+), and 40.5% (200/494) were negative (0/1+). Among equivocal cases, 28.2% (58/205) demonstrated *ERBB2* amplification, yielding an overall HER2-positive rate of 27.5% (132/480). Re-assessment with gastric scoring criteria demonstrated variability in HER2 classification, with high concordance in cytology specimens (100%) and resections (90.8%; K = 0.842, *p* < 0.001) but substantially lower concordance in biopsies (60.6%; K = 0.401, *p* < 0.001), mainly due to reclassification of equivocal cases. Notably, 47.9% (*n* = 34) of ISGyP-equivocal biopsy specimens were reclassified as HER2 IHC 3+ using gastric biopsy criteria, potentially expanding eligibility for T-DXd therapy. Conclusions: These findings highlight the evolving nature of HER2 testing in EC and demonstrate the significant impact of scoring methodology on HER2 interpretation. Our results support the development of EC-specific HER2 testing guidelines and a dual-reporting approach incorporating both ISGyP and gastric scoring criteria, with selective confirmatory FISH testing, to optimize patient selection for HER2-targeted therapies.

## Simple Summary

Some endometrial cancers produce high levels of a protein called HER2 and may benefit from HER2-targeted therapies. However, HER2 testing methods are not standardized and vary between institutions. In this study, we audited HER2 testing in 494 tumor samples from 458 patients treated at a large academic center between February 2021 and May 2025. We found that HER2 testing in endometrial cancer is complex and continues to evolve, and that the scoring method used can influence test results. These findings support the need for endometrial cancer-specific HER2 testing guidelines to ensure appropriate selection of patients for HER2-targeted therapies.

## 1. Introduction

Endometrial cancer (EC) is the most common gynecological malignancy in developed countries, with rising incidence and disease-associated mortality worldwide [1,2]. Approximately 15–20% of patients are diagnosed with high-risk disease, characterized by *TP53* mutations, high-grade non-endometrioid histology, substantial/extensive lymphovascular space invasion (LVSI), and/or advanced-stage presentation posing significant therapeutic challenges [3,4]. The identification of actionable molecular targets is therefore critical for improving outcomes.

Advances over recent years significantly enhanced our understanding of the molecular pathogenesis of EC. Notably, the Cancer Genome Atlas (TCGA)-based molecular classification of EC has demonstrated substantial prognostic value and enhanced current clinicopathological risk assessments [5,6]. This molecular classification categorizes EC into four distinct subgroups: POLE-ultramutated, mismatch repair-deficient, p53-abnormal and no-specific-molecular-profile. Prioritizing the identification of therapeutic targets within the p53-abnormal EC subgroup is of particular interest, given its poor prognosis [7,8]. Over the past several decades, human epidermal growth factor receptor 2 (HER2) has been established as a key oncogenic driver and a clinically important prognostic and predictive biomarker in breast cancer [9,10]. Extensive research has led to major advances in our understanding of the role of HER2 in tumor biology and treatment, most notably the development of HER2-targeted therapies, which have significantly improved outcomes in both early-stage and advanced disease [11,12]. These landmark studies established HER2 as a paradigm for biomarker-driven therapy and subsequently prompted evaluation of HER2 across additional tumor types, with overexpression and/or amplification most commonly observed in high-grade serous, carcinosarcoma and clear cell histologic subtypes [13]. This potential has been underscored by clinical trials demonstrating the efficacy of HER2-targeted therapies. The landmark randomized phase II trial (NCT01367002) demonstrated that adding trastuzumab to chemotherapy significantly improved progression-free and overall survival in patients with advanced/recurrent HER2-positive serous carcinomas compared to chemotherapy alone [14,15]. Notably, this trial utilized HER2 scoring criteria adapted from the 2013 American Society of Clinical Oncology/College of American Pathologists (ASCO/CAP) guidelines developed for breast cancer. In contrast, more recently, the DESTINY-PanTumor02 trial (NCT04482309), which evaluated the efficacy of the antibody-drug conjugate trastuzumab deruxtecan (T-DXd) across multiple solid tumors, utilized HER2 scoring criteria established for gastric cancer [16]. In this trial, HER2 status was based solely on immunohistochemistry (IHC), without requiring *ERBB2* amplification by in situ hybridization (ISH). Among EC patients, an objective response rate of 57.5% was reported, with the highest efficacy observed in tumors with HER2 IHC 3+ scores [16]. Subsequently, the National Comprehensive Cancer Network (NCCN) initially recommended HER2 testing in advanced or recurrent serous carcinomas and carcinosarcomas, later extending it to all TP53-mutated/p53-abnormal ECs regardless of histotype, with trastuzumab added to standard chemotherapy for HER2-positive cases. In 2024, the FDA granted tumor-agnostic accelerated approval of T-DXd for unresectable or metastatic solid tumors with HER2 IHC 3+ using gastric criteria after progression on standard therapies. NCCN guidelines now recommend T-DXd as a second-line option for recurrent ECs with IHC 3+. The evolving landscape of HER2 testing in EC has been recently reviewed elsewhere [17].

Despite these advancements, no consensus exists on standardized HER2 testing or scoring in EC and institutional practices remain highly variable [17]. This lack of standardization poses a challenge for pathologists and limits consistent patient triage for HER2-targeted therapies. Standardization of testing criteria is fundamental to optimizing patient selection and improving therapeutic outcomes.

This study aimed to review HER2 testing practices in EC at a major academic center with a reference gynecologic oncology service and biomarker laboratory, assessing HER2 status using both gynecologic- and gastric-specific scoring criteria to inform therapeutic decision-making for HER2-targeted therapies in EC. This comparative approach reflects both established gynecologic pathology practice and current scoring systems used for T-DXd eligibility assessment.

## 2. Materials and Methods

### 2.1. Study Population

This study was approved by the Sunnybrook Health Sciences Centre Research Ethics Board (SUN-5582) and written informed consent was waived. A retrospective review of the laboratory information system (Sunquest CoPath, Sunquest Information Systems, Tucson, AZ, USA) was conducted to identify ECs that underwent HER2 testing between February 2021 and January 2025.

Clinicopathological data, including patient age at diagnosis, date of primary diagnosis, tumor histological subtype and grade, estrogen receptor (ER) and progesterone receptor (PR) status, mismatch repair (MMR) protein (MLH1, MSH2, MSH6, and PMS2) immunohistochemistry (IHC) results, p53-IHC status, and available *POLE*- and *ERBB2*-mutation information, were extracted from pathology reports. Histological subtypes were confirmed by expert gynecologic pathologists through upfront pathology review prior to HER2 testing. Among biopsy material 86.9% had tumor tissue 1 cm or larger. Similarly, 93.3% of resection specimens measured greater than 1 cm. Tumor size assessment was available in 248 (50.2%) patients.

HER2 testing was typically performed on carcinosarcomas, high-grade ECs, rare advanced or recurrent low-grade endometrioid cases, and occasionally on tumors with normal/wild-type p53 expression, based on clinical requests, in accordance with institutional guidelines. Cases with ambiguous primary site of origin were excluded from this analysis. This represents a clinically selected real-world cohort enriched for high-risk EC subtypes, reflecting current clinical indications for HER2 testing. Therefore, the findings are not intended to be generalizable to an unselected EC population.

### 2.2. HER2 Assessment by Immunohistochemistry—ISGyP Criteria

HER2 IHC staining was performed on whole-slide tissue sections using Ventana anti-HER2 4B5 rabbit monoclonal antibody (Roche, Basel, Switzerland) as per current standards validated for clinical practice [18]. HER2 IHC stained slides were reviewed by one of two expert gynecologic and breast pathologists (A.P. or S.N-M.) and originally scored according to the 2020 ISGyP recommendations [19] (Table 1). Membranous staining intensity, completeness and extent were assessed on whole-slide sections. Tumors classified as equivocal (IHC 2+ scores) for HER2 protein overexpression were reflexed to FISH.

### 2.3. Re-Review of HER2 Immunohistochemistry—Gastric Scoring Criteria

For this study, the HER2 IHC slides were re-evaluated based on gastric scoring criteria, blinded to the reported score based on ISGyP recommendations (Table 1). In challenging cases a GI/molecular pathologist (W-Y.H.) was consulted as well.

### 2.4. HER2/neu Testing by Fluorescence In Situ Hybridization

Cases with equivocal IHC (score 2+) were tested by dual-probe fluorescence in situ hybridization (FISH) for HER2/neu amplification using one of two protocols that were validated independently in our clinical laboratory, depending on reagents availability affected by the COVID19 pandemic: (1) PathVysion HER-2 DNA Probe kit (Vysis, Dover, IL, USA); (2) automated HER2 IQFISH pharmDx (Dako Omnis platform, Agilent Technologies, Santa Clara, CA, USA) assay, in accordance with the current standards validated for clinical practice (Supplementary Materials). Formal concordance testing between the two validated FISH protocols was not performed. H&E, IHC and FISH slides were reviewed together for correlation.

Whole sections were evaluated by experienced HER2 FISH readers gynecologic and breast pathologists (A.P. or S.N-M.) in accordance with the 2020 ISGyP recommendations [19]. HER2 amplification (IHC 2+/ISH+) was defined by a HER2/CEP17 ratio of ≥2.0 or average HER2 copy number of ≥6 per nucleus in a tight group of minimum 20 cells. Hematoxylin and eosin (H&E), IHC and FISH slides were produced using sequential sections mounted in similar orientation and evaluated together. The entire tissue section was visualized on a fluorescent microscope at low power using DAPI filter to identify the tumor-rich areas. The entire tumor was visualized at high magnification (objective 40× and if required 60×) using the Single-Bandpass Filter Sets orange filter (designed to detect HER2-gene signals) with special attention to the areas showing the highest IHC intensity. When variations in the number of HER2-gene signals occurred, pathologists performed formal enumeration in areas with the highest signal count number. The green filter, FITC, (designed to detect CEP17 signals) was used for enumeration and computation of FISH ratio at high magnification (objective 40×). At the pathologists’ discretion, evaluation under emersion oil (objective 100×) was undertaken in selected cases. Signal counting was done in sets of at least 20 adjacent tumor cells without selective scoring, avoiding preferential count of nuclei with higher signal ratios (so called “cherry picking”). In cases with significant heterogeneity of the HER2 signal, 60 cells were counted. The FISH score was reported in the area with the highest score, and a note about heterogeneity and proportion of amplified area was added.

### 2.5. Statistical Analyses

Descriptive statistical methods were employed to summarize patient clinical characteristics and HER2 status. Cohen’s kappa coefficient was used to assess the concordance between ISGyP and Gastric HER2 scoring criteria. A 2-sided *p* < 0.05 was considered statistically significant. All statistical analyses were conducted using SPSS version 26 (IBM).

## 3. Results

### 3.1. Patient and Tumor Characteristics

From February 2021, through May 2025, a total of 494 tumor tissue samples from 458 patients were evaluated by HER2 IHC and, in cases with equivocal IHC score, FISH was performed to yield combined (IHC-ISH) HER2 status. The median age at diagnosis was 68 years (range 32–93). Briefly, specimens included 287 resections, 203 biopsies, two pleural effusions, one fine needle aspiration and one ascitic fluid sample, comprising 420 primary ECs, 27 local recurrences and 47 metastatic lesions. Serous carcinoma, either pure or as a component of mixed EC, accounted for 54.0% (267/494) of cases, while non-serous ECs comprised 46.0% (227/494). Non-serous EC histologic subtypes included high-grade, unspecified (18.2%, 90/494), carcinosarcoma (13.6%, 67/494), endometrioid carcinoma (10.1%, 50/494), clear cell carcinoma (1.4%, 7/494), gastric-type endometrial adenocarcinoma (1.0%, 5/494), undifferentiated carcinoma (0.6%, 3/494), mesonephric-like carcinoma (0.6%, 3/494) and neuroendocrine carcinoma (0.4%, 2/494). A detailed breakdown of clinicopathologic characteristics of the cohort is provided in Table 2.

### 3.2. Evaluation of HER2 IHC/FISH Using ISGyP Scoring Criteria

Using the ISGyP scoring algorithm, HER2 IHC results were as follows: 40.5% (200/494) of tumors were HER2-negative (IHC 0 or 1+), 44.5% (220/494) were equivocal (IHC 2+) and 15.0% (74/494) were positive IHC 3+. Reflex HER2 FISH testing was performed in 206 (93.6%) of 220 equivocal cases. Among these, 148 (71.8%) lacked HER2 gene amplification, while 58 of 206 (28.2%) were positive. Fourteen equivocal cases did not undergo FISH and were excluded from further analysis. The overall HER2-positive rate was defined as IHC 3+ and/or FISH amplification. This included 74 IHC 3+ cases and 58 FISH-amplified cases, for a total of 132 of 480 evaluable cases, corresponding to an overall positivity rate of 27.5%.

### 3.3. Re-Assessment of HER2 IHC Using Gastric Scoring Algorithm

HER2 IHC was re-evaluated in a subset of 318 cases using the gastric cancer scoring algorithm, which defines distinct scoring criteria for surgical and biopsy specimens. The cohort included 184 resections, 132 biopsies and two cytology specimens (one pleural effusion and one ascitic fluid sample). For the purpose of this study, each sample was scored by both biopsy and resection criteria and semi-quantitative estimated tumor surface area was assigned (≤1 cm vs. >1 cm). Among the 132 biopsy specimens, 17 cases (12.9%) were HER2-negative (IHC 0/1+), 51 (38.6%) were equivocal (IHC 2+), and 64 (48.5%) were positive (IHC 3+). Of the 184 resection specimens, 37 cases (20.1%) were HER2-negative, 102 (55.4%) were equivocal, and 45 (24.5%) were positive.

Comparison of HER2 IHC scores between the biopsy and resection scoring algorithms showed concordant results in 140 (44.0%) of 318 cases. Among the discordant cases, two cases (0.6%) were scored as equivocal (2+) using biopsy criteria but were negative (0/1+) using resection criteria; both tumors measured >1 cm. In 11 cases (3.5%) the score was equivocal (2+) by biopsy criteria but as 1+ by resection scoring criteria; 10 of these tumors were >1 cm. Additionally, 31 cases (9.7%) were interpreted as positive (3+) using biopsy criteria but were equivocal (2+) using resection scoring criteria; 29 of these measured >1 cm.

### 3.4. Comparison of ISGyP to Gastric Scoring Criteria

**Figure 1:**
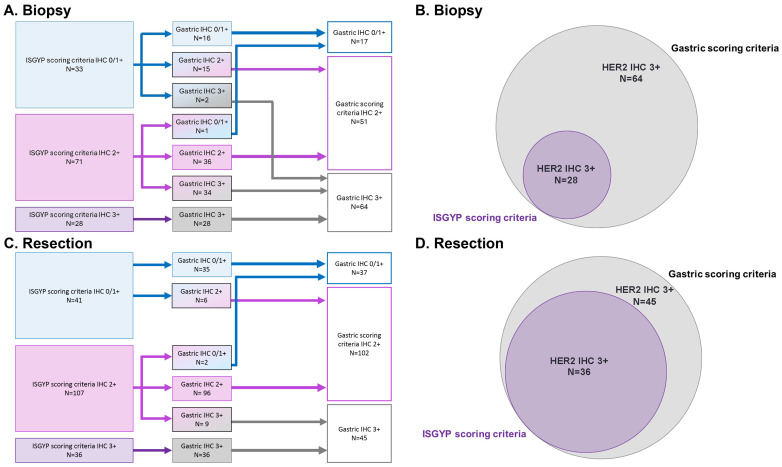
Summary of HER2 IHC scoring using ISGYP and gastric scoring criteria in biopsy and resection specimens. (**A**) Biopsy specimens. Flow diagram showing the distribution of cases when HER2 immunohistochemistry (IHC) scores assigned using ISGYP scoring criteria were reclassified according to gastric biopsy and resection scoring criteria. Arrows indicate the transition of cases between categories. Blue boxes represent IHC 0/1+, pink boxes represent IHC 2+, and gray boxes represent IHC 3+. The total number of cases classified as HER2 IHC 0/1+, 2+, and 3+ using gastric criteria is shown on the right. (**B**) Biopsy specimens—HER2 IHC 3+ comparison. Venn diagram illustrating the overlap of HER2 IHC 3+ cases identified by gastric scoring criteria and ISGYP scoring criteria in biopsy samples. (**C**) Resection specimens. Flow diagram showing the redistribution of HER2 IHC scores when cases initially scored using ISGYP criteria were reassessed using gastric biopsy and resection scoring criteria. Arrows represent case transitions between categories. Color coding follows the same scheme as in panel A. (**D**) Resection specimens—HER2 IHC 3+ comparison. Venn diagram demonstrating the overlap of HER2 IHC 3+ cases identified by gastric versus ISGYP scoring criteria in resection specimens.

**Figure 2:**
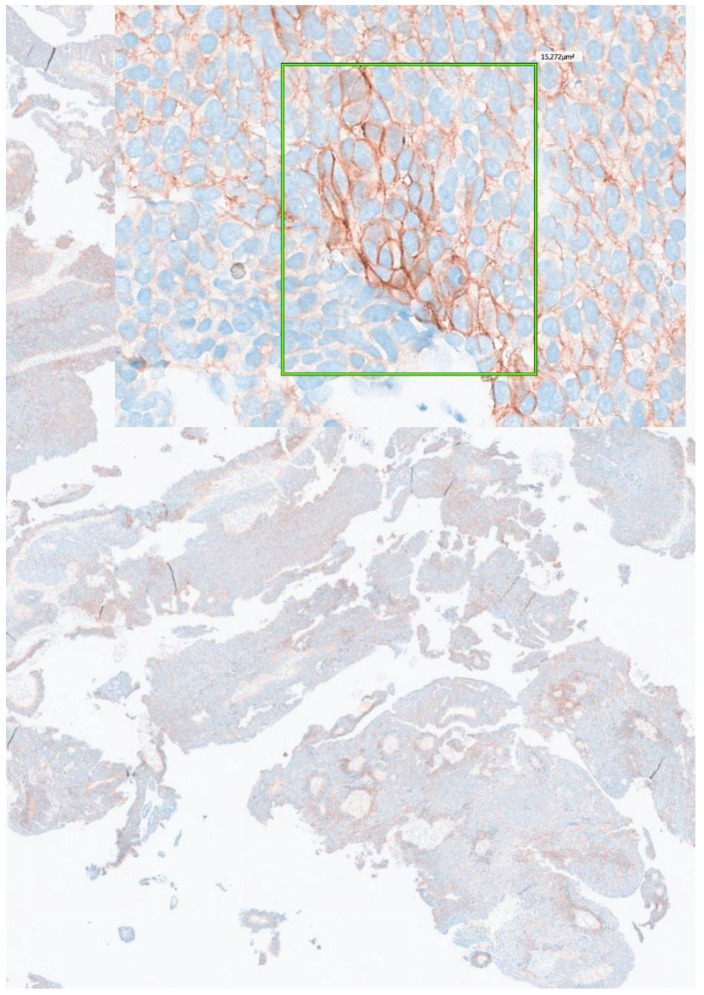
Endometrial biopsy: high-grade serous carcinoma (p53 null). HER2 immunohistochemistry is scored 2+ using ISGyP criteria. Green insert: A rare cluster with complete strong membrane labeling is noted (>5 cells) warrants score as 3+ by gastric criteria for biopsy specimen is applied. FISH analysis is negative (Ratio = 1).

**Figure 3:**
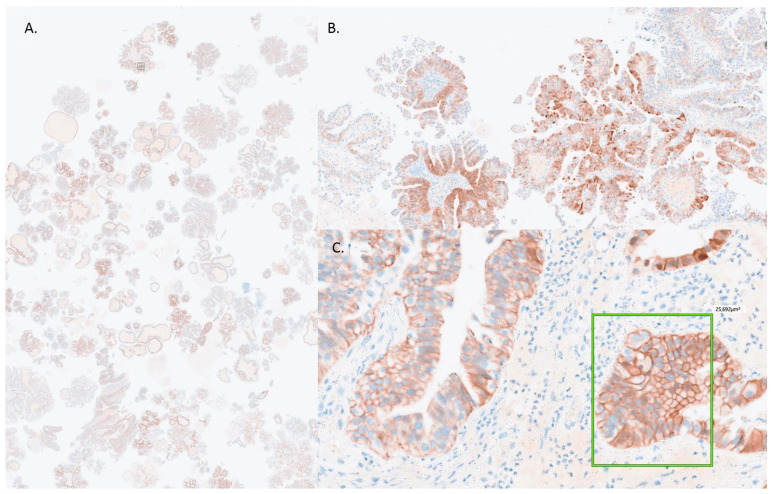
Endometrial biopsy: high-grade serous carcinoma (p53 overexpressed). (**A**) HER2 immunohistochemistry is scored 2+ using ISGyP criteria. (**B**) Most of the labeling appreciated on lower power represents non-specific cytoplasmic blush. (**C**) Green insert: Cluster with complete strong membrane labeling is noted (>5 cells) and warrants score as 3+ by gastric criteria for biopsy specimen. FISH analysis is positive (Ratio = 3).

**Figure 4:**
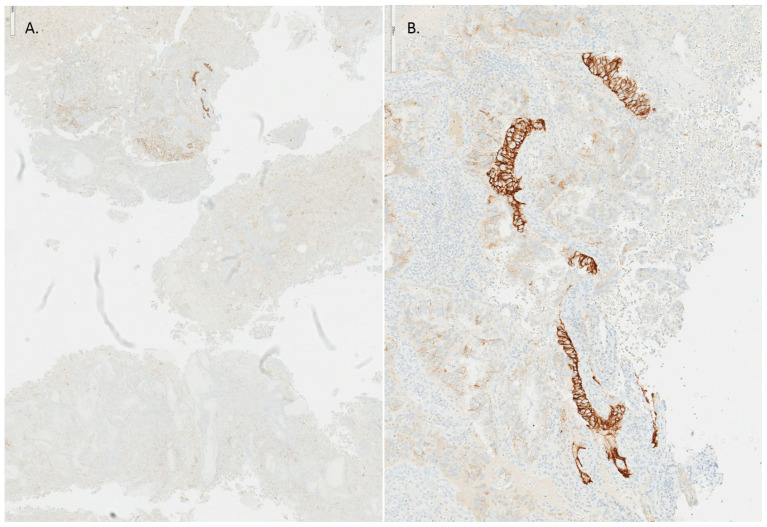
Endometrial biopsy: high-grade serous carcinoma (p53 overexpressed). (**A**) HER2 immunohistochemistry is highly heterogenous, scored 2+ using ISGyP criteria. (**B**) Few clusters with complete strong membrane labeling are noted (>5 cells) and warrant 3+ score by gastric criteria for biopsy specimen. FISH analysis is positive (Ratio = 8 in these clusters).

Comparison of HER2 IHC results using ISGyP versus gastric cancer scoring algorithms demonstrated variable concordance across specimen types (Table 3, Figure 1). For biopsies (N = 132), overall concordance was 60.6% (80/132; K = 0.401, *p* < 0.001). Of the IHC positive tumors using ISGyP criteria (IHC 3+, *n* = 28), all were also positive using gastric biopsy criteria. Among the equivocal cases on ISGyP criteria (*n* = 71), 36 (50.7%) were concordantly scored as equivocal (2+), whereas one (1.4%) was negative (IHC 0/1+) and 34 (47.9%) were positive (IHC 3+) using gastric biopsy criteria (Figure 2, Figure 3 and Figure 4). Of the ISGyP-negative tumors (*n* = 33), 16 (48.5%) remained negative using gastric biopsy scoring criteria, while 15 (45.5%) were classified as equivocal and two (6.1%) as positive.

For resections (N = 184), concordance was substantially higher, with 90.8% (167/184) of cases in agreement (K = 0.842, *p* < 0.001). All ISGyP-positive tumors (IHC 3+, *n* = 36, 100%) were also positive by gastric resection criteria. Among the ISGyP-equivocal cases (IHC 2+, *n* = 107), 96 (89.7%) were concordantly scored as equivocal, two (1.9%) were negative and nine (8.4%) were positive by gastric resection criteria. Of the ISGyP-negative tumors (IHC 0/1+, *n* = 41), 35 (85.4%) remained negative by gastric resection scoring criteria, while six (14.6%) were reclassified as equivocal. Cytology specimens (N = 2) demonstrated complete concordance (100%), with both classified as positive (IHC 3+) by both ISGyP and gastric scoring criteria.

## 4. Discussion

This large single-institution study evaluated HER2 testing practices in EC using both ISGyP and gastric cancer-specific scoring algorithms. It represents a unique experience in which pathologists with expertise in breast and gynecologic oncology interpreted whole-slide HER2 FISH assays. In contrast, most previously reported studies have relied on tissue microarray (TMA)-based FISH analyses interpreted by molecular pathologists or cytogeneticists, who commonly perform and report FISH testing in clinical laboratories.

Using ISGyP criteria, the overall HER2 positivity rate, defined as IHC 3+ and/or FISH amplification, was 27.5%. Reassessment with gastric scoring criteria revealed substantial variability in HER2 classification depending on whether biopsy or resection-specific criteria were applied. Concordance between ISGyP and gastric scoring criteria was high in cytology specimens (100%) and resections (90.8%), but substantially lower in biopsies (60.6%), primarily due to reclassification of equivocal cases.

Gastric biopsy criteria define HER2 positivity (IHC 3+) as strong, complete basolateral or lateral membrane staining in clusters of ≥5 tumor cells, regardless of the overall percentage of positive tumor cells. While this approach is practical for gastric cancer, where diagnostic biopsies are often limited in size, its applicability to endometrial curettage and/or biopsy specimens, typically yielding larger tissue fragments, warrants further assessment. The clinical rationale for applying gastric criteria in this setting comes from the DESTINY-PanTumor02 trial (NCT04482309) [16], in which the strongest activity of T-DXd in EC was observed in HER2 3+ tumors. Therefore, use of gastric criteria may increase sensitivity for tumor’s eligibility for T-DXd. However, in EC specimens, this increased sensitivity should be balanced against the risk of overcalling a small cluster with HER2 expression within heterogeneous tumors. In our cohort, 86.9% of biopsy specimens contained tumor tissue measuring ≥1 cm, underscoring that endometrial biopsies typically provide adequate material for evaluation. In these larger curettage/biopsy specimens, small clusters of tumor cells may fulfill the gastric IHC 3+ threshold despite overall heterogeneous or focal HER2 expression, raising questions regarding the predictive value of such staining patterns for therapeutic response [18,20,21]. Notably, patients with HER2 2+ EC also derived benefit in the DESTINY-PanTumor02 trial, suggesting that the potential for overtreatment may be limited in this context [16]. But this data does not eliminate the need for caution when classifying tumors as IHC3+ based on focal staining alone. In our study, 34 (47.9%) of ISGyP-equivocal biopsy tumors were reclassified as positive HER2 3+ using gastric criteria, allowing for consideration of T-DXd therapy.

Several challenges in HER2 scoring for rare patterns remain unresolved. Gastric scoring criteria does not explicitly address how to score biopsy specimens containing fewer than five HER2 IHC 3+ tumor cells. Clarity is also needed regarding the interpretation of strong or moderate staining present in ≤10% of tumor cells under either ISGyP or gastric criteria. Further, the appropriate classification of surgical specimens demonstrating a small cluster of strongly positive (HER2 IHC 3+) tumor cells comprising <10% of the tumor, when assessed using gastric criteria, is not clearly defined in current reporting guidelines. Although ISH is not required for biopsy specimens evaluated using gastric criteria, we have encountered cases with small clusters of five tumor cells that were not amplified by ISH, underscoring the need for explicit clarification.

Notably, current evidence suggests that HER2-low and HER2-ultra-low status may not have the same biological or therapeutic relevance in EC as in other tumor types, and therefore reporting these categories is not currently recommended [22].

Although comparison between HER2 IHC and *ERBB2* FISH status was beyond the scope of this manuscript, discordance between HER2 protein expression and gene amplification has been previously reported in EC and is also reflected in our experience [21,23,24]. *ERBB2* amplification without corresponding protein overexpression has been described in rare cases and may reflect intratumoral heterogeneity, sampling effects or technical and interpretative variability in IHC scoring [21,23,24,25]. Tumor heterogeneity may result in coexistence of amplified and non-amplified tumor clones withing the same lesion. Sampling effect may contribute because IHC and FISH often assess non-identical foci from deeper sections. Different methodologies cutoffs may accentuate the discordance. For example, under gastric biopsy criteria a small cluster of tumor cells may be sufficient for classification as HER2 3+. However, the corresponding focus may not be present on deeper sections used for FISH. Moreover, even when present, precise localization of the same small cluster during FISH assessment may be challenging. Bright-field ISH may help overcome this limitation by improving morphologic correlation.

Conversely, *ERBB2* amplification without corresponding protein overexpression has been described in rare cases, likely due to intratumoral heterogeneity or technical and interpretative variability in IHC scoring, further underscoring the imperfect correlation with FISH in EC [21,23,24,25]. In our practice, whole-slide FISH assessment may improve detection of heterogeneity compared with limited-area approaches. While HER2 3+/FISH-negative cases may raise uncertainty regarding predictive response to HER2-directed therapy, current clinical trials and treatment algorithms predominantly rely on IHC-based classification given its low cost and broad availability. Accordingly, further studies correlating *ERBB2* status and HER2 protein expression and clinical outcomes are warranted to refine patient selection.

A key limitation of this retrospective single-institution pathology audit is lack of treatment-response and survival data, preventing assessment of the predictive value of either HER2 scoring system. Therefore, prospective studies with integrated clinical outcome data are needed to determine whether the observed reclassification translates into differences in therapeutic response and patient outcomes. In addition, the cohort represents a clinically selected population enriched for high-risk EC subtypes in which HER2 testing is preferentially performed according to current clinical guidelines, which may introduce a selection bias and limit generalizability to an unselected EC population. The inclusion of primary tumors, recurrent disease and metastatic sites further introduces biological heterogeneity that may influence HER2 status interpretation.

The findings in our study underscore the need for comprehensive EC-specific scoring criteria that are applicable to patient’s management and highlight the importance of reporting both scoring systems in jurisdictions where trastuzumab and T-DXd are approved for treatment. Although there are studies that have explored clinical response, suggesting that HER2 heterogeneity may contribute to trastuzumab resistance [20], comparative studies correlating HER2 scoring with clinical response to HER2-targeted therapies are still needed to determine whether current scoring approaches accurately identify patients that would benefit from treatment or inadvertently overestimates HER2 positivity. Since pathologists are not typically aware of the planned HER2 targeted therapy, reporting HER2 using both gastric and ISGyP criteria with ISH upon request is recommended.

## 5. Conclusions

In conclusion, our audit is retrospective and did not assess clinical response, as many patients whose samples are tested in our central laboratory are treated at other institutions. Nevertheless, this large single-institution study emphasizes that HER2 testing in EC remains an evolving and complex field. Our analyses highlight the impact of scoring criteria on HER2 interpretation and support a dual-reporting approach that incorporates both ISGyP and gastric scoring systems with possible confirmatory FISH testing when clinically relevant. These findings highlight the importance of standardization of HER2 testing in EC with specific HER2 testing recommendations that incorporate specimen type, heterogeneity and intended therapeutics to guide clinical decision-making in the era of expanding HER2-targeted treatment options [26].

## Figures and Tables

**Table 1:** Endometrial carcinoma HER2 scoring recommendations: 2020 ISGyP guidelines for trastuzumab eligibility and gastric biopsy/resection criteria for trastuzumab deruxtecan eligibility.

Immunohistochemistry (IHC)			Fluorescence In Situ Hybridization (FISH)	
Score	Criteria	Quantitative Threshold	Category	Recommendation
2020 ISGyP Guidelines for HER2 Testing for Trastuzumab Eligibility for biopsy/curettage and resection specimens *				
0	No staining observed	None	Not recommended	
1+	Faint/barely perceptible, incomplete membrane staining seen in any proportion, or weak complete staining	<10% tumor cells		
2+	Weak–moderate membranous staining	≥10% of tumor cells	Positive	HER2/CEP17 ratio of ≥2.0 or average HER2 copy number of ≥6 per nucleus in a tight group of minimum 20 cells
	Overall staining extent	≤30% of tumor cells	Negative	HER2/CEP17 ratio < 2.0 and average HER2 copy number < 6/nucleus
3+	Strong basolateral/lateral or circumferential membranous staining	>30% tumor cells	Not recommended	
***Gastric Biopsy Criteria for Trastuzumab Deruxtecan Eligibility***				
0	No staining observed	None	Not recommended	
1+	Faint/barely perceptible membrane staining in any proportion	Cluster of ≥5 tumor cells (irrespective of % positive tumor cells)		
2+	Weak-to-moderate, complete, basolateral or lateral membrane staining			
3+	Strong, complete, basolateral, or lateral membrane staining			
***Gastric Resection Criteria for Trastuzumab Deruxtecan Eligibility***				
0	No staining or membranous staining (focal)	<10% tumor cells	Not recommended	
1+	Faint/barely perceptible, incomplete membranous staining	≥10% tumor cells		
2+	Weak-to-moderate, complete, basolateral/lateral membranous staining			
3+	Strong, complete, basolateral/lateral membranous staining			

* The 2020 ISGyP HER2 scoring criteria are independent of specimen type and are applied equally to biopsy/curettage and resection material.

**Table 2:** Clinical and pathologic characteristics of endometrial cancer patients tested for HER2 status.

	N (%)
**Age** (range, years)	68 (32–92)
**Specimen Type**	
Biopsy	203 (41.1)
Resection	287 (58.1)
Cytology	4 (0.8)
**Tumor type**	
Primary	420 (85.0)
Local recurrence	27 (5.5)
Metastasis	47 (9.5)
**Histologic type**	
Serous	244 (49.4)
Mixed (with serous component)	23 (4.7)
Carcinosarcoma	67 (13.6)
High-grade, unspecified	90 (18.2)
Endometrioid	50 (10.1)
Clear cell	7 (1.4)
Endometrial gastric-type carcinoma	5 (1.0)
Endometrial mesonephric-like carcinoma	3 (0.6)
Undifferentiated	3 (0.6)
Neuroendocrine carcinoma	2 (0.4)
**ER IHC**	
Positive	260 (75.4)
Negative	85 (24.6)
N/A	149
**MMR IHC**	
Intact	377 (95.9)
Deficient	14 (3.6)
Subclonal loss	2 (0.5)
N/A	101
**p53 IHC**	
Aberrant	366 (88.6)
Wild type	39 (9.4)
Equivocal	1 (0.2)
Subclonal	7 (1.7)
N/A	81

N/A, Not Applicable.

**Table 3:** Concordance between ISGyP and gastric scoring criteria for HER2 immunohistochemistry across specimen types.

Specimen Type	Gastric Scoring Criteria	ISGYP Scoring Criteria		
		Negative (0/1+)	Equivocal (2+)	Positive (3+)
Biopsy (N = 132)	Negative (0/1+)	16 (48.5%)	1 (1.4%)	0
	Equivocal (2+)	15 (45.5%)	36 (50.7%)	0
	Positive (3+)	2 (6.1%)	34 (47.9%)	28 (100%)
Cytology (N = 2)	Negative (0/1+)	-	-	-
	Equivocal (2+)	-	-	-
	Positive (3+)	-	-	2 (100%)
Resection (N = 184)	Negative (0/1+)	35 (85.4%)	2 (1.9%)	0
	Equivocal (2+)	6 (14.6%)	96 (89.7%)	0
	Positive (3+)	0	9 (8.4%)	36 (100%)

## Data Availability

The data that support the findings of this study are available on request from the corresponding author, A.P.

## Supplementary Materials

The following supporting information can be downloaded at: https://www.mdpi.com/article/10.3390/cancers18122009/s1, File S1: Protocol for HER2 IHC; Protocol for HER2 ISH.

## References

[1] Henley S.J., Ward E.M., Scott S., Ma J., Anderson R.N., Firth A.U., Thomas C.C., Islami F., Weir H.K., Lewis D.R. (2020). Annual report to the nation on the status of cancer, part I: National cancer statistics. Cancer.

[2] Bray F., Laversanne M., Sung H., Ferlay J., Siegel R.L., Soerjomataram I., Jemal A. (2024). Global cancer statistics 2022: GLOBOCAN estimates of incidence and mortality worldwide for 36 cancers in 185 countries. CA Cancer J. Clin..

[3] Lu K.H., Broaddus R.R. (2020). Endometrial Cancer. N. Engl. J. Med..

[4] de Boer S.M., Powell M.E., Mileshkin L., Katsaros D., Bessette P., Haie-Meder C., Ottevanger P.B., Ledermann J.A., Khaw P., Colombo A. (2018). Adjuvant chemoradiotherapy versus radiotherapy alone for women with high-risk endometrial cancer (PORTEC-3): Final results of an international, open-label, multicentre, randomised, phase 3 trial. Lancet Oncol..

[5] Kandoth C., Schultz N., Cherniack A.D., Akbani R., Liu Y., Shen H., Robertson A.G., Pashtan I., Shen R., Cancer Genome Atlas Research Network (2013). Integrated genomic characterization of endometrial carcinoma. Nature.

[6] Talhouk A., McConechy M.K., Leung S., Li-Chang H.H., Kwon J.S., Melnyk N., Yang W., Senz J., Boyd N., Karnezis A.N. (2015). A clinically applicable molecular-based classification for endometrial cancers. Br. J. Cancer.

[7] Leon-Castillo A., de Boer S.M., Powell M.E., Mileshkin L.R., Mackay H.J., Leary A., Nijman H.W., Singh N., Pollock P.M., Bessette P. (2020). Molecular Classification of the PORTEC-3 Trial for High-Risk Endometrial Cancer: Impact on Prognosis and Benefit from Adjuvant Therapy. J. Clin. Oncol..

[8] Chang Y.W., Kuo H.L., Chen T.C., Chen J., Lim L., Wang K.L., Chen J.R. (2024). Abnormal p53 expression is associated with poor outcomes in grade I or II, stage I, endometrioid carcinoma: A retrospective single-institute study. J. Gynecol. Oncol..

[9] Slamon D.J., Clark G.M., Wong S.G., Levin W.J., Ullrich A., McGuire W.L. (1987). Human breast cancer: Correlation of relapse and survival with amplification of the HER-2/neu oncogene. Science.

[10] Dawood S., Broglio K., Buzdar A.U., Hortobagyi G.N., Giordano S.H. (2010). Prognosis of women with metastatic breast cancer by HER2 status and trastuzumab treatment: An institutional-based review. J. Clin. Oncol..

[11] Lewis Phillips G.D., Li G., Dugger D.L., Crocker L.M., Parsons K.L., Mai E., Blattler W.A., Lambert J.M., Chari R.V., Lutz R.J. (2008). Targeting HER2-positive breast cancer with trastuzumab-DM1, an antibody-cytotoxic drug conjugate. Cancer Res..

[12] Geyer C.E., Forster J., Lindquist D., Chan S., Romieu C.G., Pienkowski T., Jagiello-Gruszfeld A., Crown J., Chan A., Kaufman B. (2006). Lapatinib plus capecitabine for HER2-positive advanced breast cancer. N. Engl. J. Med..

[13] Buza N., Roque D.M., Santin A.D. (2014). HER2/neu in Endometrial Cancer: A Promising Therapeutic Target with Diagnostic Challenges. Arch. Pathol. Lab. Med..

[14] Fader A.N., Roque D.M., Siegel E., Buza N., Hui P., Abdelghany O., Chambers S.K., Secord A.A., Havrilesky L., O’Malley D.M. (2018). Randomized Phase II Trial of Carboplatin-Paclitaxel Versus Carboplatin-Paclitaxel-Trastuzumab in Uterine Serous Carcinomas That Overexpress Human Epidermal Growth Factor Receptor 2/neu. J. Clin. Oncol..

[15] Fader A.N., Roque D.M., Siegel E., Buza N., Hui P., Abdelghany O., Chambers S., Secord A.A., Havrilesky L., O’Malley D.M. (2020). Randomized Phase II Trial of Carboplatin-Paclitaxel Compared with Carboplatin-Paclitaxel-Trastuzumab in Advanced (Stage III–IV) or Recurrent Uterine Serous Carcinomas that Overexpress Her2/Neu (NCT01367002): Updated Overall Survival Analysis. Clin. Cancer Res..

[16] Meric-Bernstam F., Makker V., Oaknin A., Oh D.Y., Banerjee S., Gonzalez-Martin A., Jung K.H., Lugowska I., Manso L., Manzano A. (2024). Efficacy and Safety of Trastuzumab Deruxtecan in Patients with HER2-Expressing Solid Tumors: Primary Results from the DESTINY-PanTumor02 Phase II Trial. J. Clin. Oncol..

[17] Buza N. (2025). The Rapidly Evolving Landscape of Human Epidermal Growth Factor Receptor 2 (HER2) Testing in Endometrial Carcinoma and Other Gynecologic Malignancies. Arch. Pathol. Lab. Med..

[18] Plotkin A., Olkhov-Mitsel E., Huang W.Y., Nofech-Mozes S. (2024). Implementation of HER2 Testing in Endometrial Cancer, a Summary of Real-World Initial Experience in a Large Tertiary Cancer Center. Cancers.

[19] Buza N. (2021). HER2 Testing and Reporting in Endometrial Serous Carcinoma: Practical Recommendations for HER2 Immunohistochemistry and Fluorescent In Situ Hybridization: Proceedings of the ISGyP Companion Society Session at the 2020 USCAP Annual Meeting. Int. J. Gynecol. Pathol..

[20] Shen S., Ma W., Brown D., Da Cruz Paula A., Zhou Q., Iaosonos A., Tessier-Cloutier B., Ross D.S., Troso-Sandoval T., Reis-Filho J.S. (2023). HER2 Genetic Intratumor Heterogeneity Is Associated with Resistance to Trastuzumab and Trastuzumab Emtansine Therapy in Recurrent High-Grade Endometrial Cancer. Mod. Pathol..

[21] Salinaro J., Singh K., Sands N., Gill V., Perati S., James N., Sharma S., Nasir A., DiSilvestro P., Miller K. (2025). Distribution and concordance of HER2 scores in endometrial and ovarian cancer. Gynecol. Oncol..

[22] Cortes-Salgado A., Moreno-Moreno E., Carretero-Barrio I., Caniego-Casas T., Cristobal E., Del Campo-Albendea L., Guerra E., Alia V., de Aguado P.P., Corraliza V. (2025). HER2 expression in a molecularly defined cohort of endometrial cancer patients: The SPECTRUM study. Gynecol. Oncol..

[23] Klc T.R., Wu S., Wilhite A.M., Jones N.L., Powell M.A., Olawaiye A., Girda E., Brown J., Puechl A., Ali-Fehmi R. (2022). HER2 in Uterine Serous Carcinoma: Testing platforms and implications for targeted therapy. Gynecol. Oncol..

[24] Aickin C.C., Brading A.F. (1985). Advances in the understanding of transmembrane ionic gradients and permeabilities in smooth muscle obtained by using ion-selective micro-electrodes. Experientia.

[25] Menshikova E., Deeb K., Genega E.M., Hanley K., Turashvili G. (2025). Analysis of Human Epidermal Growth Factor Receptor 2 (HER2) Heterogeneity at the Protein and Gene Levels in Endometrial Cancer: Refining HER2 Reporting and HER2-Directed Therapies. Lab. Investig..

[26] Turashvili G., Karnezis A.N., Hulkower K.I., Hebert C., Harik L., Crothers B., Giannico G., Deeb K.K., Hanley K., Ganesan R. (2025). Reporting Results of Biomarker Testing of Specimens from Patients with Carcinoma of Gynecologic Origin: The Updated College of American Pathologists Protocol. Arch. Pathol. Lab. Med..

